# Comparing trends in mortality from cardiovascular disease and cancer in the United Kingdom, 1983–2013: joinpoint regression analysis

**DOI:** 10.1186/s12963-017-0141-5

**Published:** 2017-07-01

**Authors:** Lauren Wilson, Prachi Bhatnagar, Nick Townsend

**Affiliations:** Nuffield Department of Public Health, British Heart Foundation Centre on Population Approaches for Non-Communicable Disease Prevention, University of Oxford, Old Road Campus, Oxford, OX3 7LF UK

**Keywords:** Cancer, Cardiovascular disease, Epidemiology, Mortality, Joinpoint regression analysis

## Abstract

**Background:**

We aimed to study the time trends underlying a change from cardiovascular disease (CVD) to cancer as the most common cause of age-standardized mortality in the UK between 1983 and 2013.

**Methods:**

A retrospective trend analysis of the World Health Organization mortality database for mortality from all cancers, all CVDs, and their three most common types, by sex and age. Age-standardized mortality rates were adjusted to the 2013 European Standard Population and analyzed using joinpoint regression analysis for annual percent changes.

**Results:**

The difference in mortality rate between total CVD and cancer narrowed over the study period as age-standardized mortality from CVD decreased more steeply than cancer in both sexes. We observed higher overall rates for both diseases in men compared to women, with high mortality rates from ischemic heart disease and lung cancer in men. Joinpoint regression analysis indicated that trends of decreasing rates of CVD have increased over time while decreasing trends in cancer mortality rates have slowed down since the 1990s. The lowest improvements in mortality rates were for cancer in those over 75 years of age and lung cancer in women.

**Conclusions:**

In 2011, the age-standardized mortality rate for cancer exceeded that of CVD in both sexes in the UK. These changing trends in mortality may support evidence for changes in policy and resource allocation in the UK.

## Background

In 2013, cardiovascular disease (CVD) was the most common cause of mortality in Europe, Asia, and the Americas, with the majority of CVD deaths occurring due to ischemic heart disease (IHD) [1]. Cancer was the second most common cause of death in these regions, with lung cancer the most common type. CVD had been the most common cause of death in the UK since the middle of the twentieth century but was overtaken by cancer in 2011 for men [2]. Of the 570,341 deaths in the United Kingdom (UK) in 2014, 29.4% were due to cancer and 27.1% were due to CVD, with both diseases still representing major public health burdens.

Recent publications suggest a change from CVD to cancer as the most common cause of death in the UK and some other European countries [3, 4]. This warrants a comparative analysis as both diseases have been decreasing in the UK since the 1980s. In particular, IHD mortality in the UK had one of the largest decreases in Europe [5]. Recent articles have suggested that this decrease is not equal across population groups, with exceptions such as a plateau in decreasing CVD mortality at younger ages [3, 6] and a rise in female but not male lung cancer mortality [4].

It is unclear how underlying sex-, age-, and disease-type-specific trends have contributed toward a shift from CVD to cancer as the most common cause of death and when such changes in mortality rates occurred. We found only one study from Portugal that compared cancer and CVD mortality within the same analysis for a European country [7]. Although their study considered trends in age-standardized mortality rates and years of life lost by age group and sex from 1980 to 2010, their breakdown was limited to total CVD and total cancer rates. The analysis of disease types is important due to the differences in etiology between CVD and cancer types [8]. For this reason, our analysis includes mortality trends in IHD and stroke that account for 71% of UK CVD deaths, and the four leading causes of cancer mortality (lung, colon, breast, and prostate) which account for 45% of UK cancer mortality. We aimed to compare observational trends in CVD and cancer mortality by sex, age, and disease types from 1983 to 2013, to provide evidence for recent UK mortality trends.

## Data and methods

### Mortality data

We obtained the number and cause of medically certified deaths for the United Kingdom from the World Health Organization (WHO) global mortality database online (updated November 2015). Data were available for all calendar years from 1983 to 2013, except the year 2000, grouped by five-year age groups and sex. Data between the years 1983 and 1999 used the International Classification of Diseases (ICD) revision 9 up to 85+ years of age. Years 2001 to 2013 used the ICD 10th revision up to age 95+ years of age. We chose disease categories based on the leading causes of death in the UK and ICD − 10 categories as utilized by WHO and the Global Burden of Disease study [9, 10]. Data for total deaths were obtained for Chapter 2 (neoplasms) and Chapter 9 (circulatory system) of the ICD and their three most common disease types in the UK (Table 1). Concordant coding was used to match transition between ICD revisions [10]. We included benign tumors in the broad analysis due to coding reassignments from non-malignant to malignant, and vice versa, from the revision of ICD-9 to ICD-10 in the UK [9]. We excluded benign tumors from type-specific analysis due to lack of accessible data before 2001. Western Europe refers to the definition used by the Global Burden of Disease study.

**Table 1:** International Classification of Disease (ICD) by revision and years utilized in the UK, 1983–2013

WHO ICD definition	Detailed ICD-10 2001–2013^a^	Detailed ICD-9 1998–1999	ICD-9 basic tabulation 1983–1997
Neoplasms			
Malignant	C00-C97	140–208	B08-B14
Benign, in situ*,* or unknown	D00-D48	210–239	B15-B17
Trachea, bronchus, lung	C33, C34	162	B101
Colon, sigmoid, rectum, anus	C18-C21	153, 154	B093, B094
Breast (female)	C50	174	B113
Prostate (male)	C61	185	B124
Diseases of the circulatory system	I00-I99	390–459	B25-B30
Ischemic heart diseases Cerebrovascular diseases Other cardiovascular & heart disease	I20-I25 I60-I69 I00, I26-I28, I34-I37, I44-I51, I70-I99	410–414 430–438 415–417, 423–424, 426–429, 440–448, 451–459	B27 B29 B28, B30

^a^ Year 2000 data not available

### Population data

We obtained midyear population estimates for the UK from the Office for National Statistics (ONS) website. We used these in preference to WHO data as this source provided the most up-to-date population estimates from the 2011 census. Data were available for each calendar year in five-year age groups up to 85+ years until 2000, and up to 90+ years thereafter.

### Age-standardization

Mortality and population data were organized into five-year age groups, up to 85+ years, to correspond with age categories used in the 2013 European Standard Population (ESP) and those provided in mortality and population data. We calculated age-standardized rates per 100,000 with 95% confidence intervals (CI) for each study year (1983–2013) using the direct method, based on the 2013 ESP and age-specific crude rates. Microsoft Excel 2010 was used to calculate age-standardized rates by sex and age groups.

### Annual percentage change (APC)

We used joinpoint regression to analyze trends in age-standardized CVD and cancer mortality rates. Analysis was performed by sex, 15-year age group, and disease type. Joinpoint analysis identifies the best fit for inflexion points (“joinpoints”) at which there is a significant change in trends using a series of permutation tests, with Bonferroni adjustment for multiple comparisons. In this study, joinpoint analysis was used to identify years (as the independent variable) at which significant changes in mortality rate occurred over the study period and the size of these changes (as the percentage change in rate per year). The use of a natural log-linear model enables the analysis of a constant percentage change in rate over time. We allowed up to four joinpoints utilizing a Monte Carlo permutation method and evaluated whether there was a difference from no change of each segment using a *p*-value of less than 0.05 as statistically significant. We computed joinpoints using the Joinpoint trend analysis software from the Surveillance Research Program of the National Cancer Institute Version 4.2.0.2 (Statistical Research and Applications Branch, National Cancer Institute, US).

## Results

**Fig. 1:**
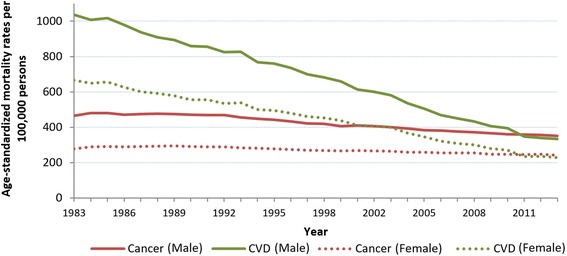
Age-standardized mortality trends for cancer (including benign) and cardiovascular diseases in the UK. Legend: Years 1983 to 2013

Of the 576,458 deaths in the UK in 2013, cancer was the recorded cause for 165,875 people and CVD was the recorded cause for 159,795 (Table 2). Between 1983 and 2013, age-standardized mortality rates for total CVDs and total cancers decreased; however, the decrease in cancer was much less steep in comparison to CVD (Fig. 1). Overall, total age-standardized CVD mortality decreased by 67% and total cancer mortality decreased by 18% over 30 years. The absolute number of deaths from cancer increased over the study period while the number of deaths from CVD decreased (Table 2).

**Table 2:** Joinpoint analysis of age-standardized^b^ mortality rate from cancer and cardiovascular diseases (CVD) by sex

	Absolute deaths (Rate per 100,000)		Total study period^c^		Period 1		Period 2		Period 3	
	1983	2013	Average APC (%)	95%CI	Years	APC (%)	Years	APC (%)	Years	APC (%)
Cancer										
Male	80,553 (464.9)	87,511 (350.9)	-1.0^a^	−1.1, −0.8	1983–1991	0.0	1991–1997	−1.8^a^	1997–2013	−1.2^a^
Female	71,265 (277.3)	78,331 (242.7)	−0.5^a^	−0.6, −0.4	1983–1989	0.7^a^	1989–1997	−1.0^a^	1997–2013	−0.7^a^
CVD										
Male	158,944 (1035.3)	79,935 (334.3)	−3.9^a^	−4.0, −3.7	1983–2001	−2.8^a^	2001–2013	−5.4^a^		
Female	164,349 (665.8)	79,860 (227.9)	−3.7^a^	−3.8, −3.5	1983–2003	−2.7^a^	2003–2013	−5.6^a^		

^a^Significantly different from zero (*p* < 0.05)

^b^ Standardized to the 2013 European standard population by 5-year age groups

^c^ Years 1983 to 2013

*APC*, annual percentage change

The decreases in age-standardized mortality rates for cancer and CVD were similar when comparing rates between sexes, but rates were consistently higher in men than in women throughout the period (Fig. 1, Table 2). The ratio of age-standardized deaths from CVD to cancer in men changed from 2.2 in 1983 to 0.95 in 2013. In women, this ratio changed from 2.4 in 1983 to 0.94 in 2013. For absolute deaths, cancer overtook CVD as the most common cause of death for men in 2011, and women in 2014 [11], but by age-standardized mortality, rates of cancer surpassed CVD in both sexes in 2011.

Joinpoint regression analysis showed one joinpoint for age-standardized CVD mortality trends in men and women, indicating two significant periods of decreasing mortality rates (Table 2). The steepest CVD decreases were in the second period, with a significant decrease of 5.4% per year in men and 5.6% in women. Trends in age-standardized cancer mortality showed two joinpoints for both sexes, with significant decreases in the second and third periods. The steepest decrease for cancer was in the second period, while the first period showed no significant change in men and a small significant increase in women. From 1983 to 2013, CVD mortality had steeper decreases than cancer in all periods.

### Age group mortality trends

**Fig. 2:**
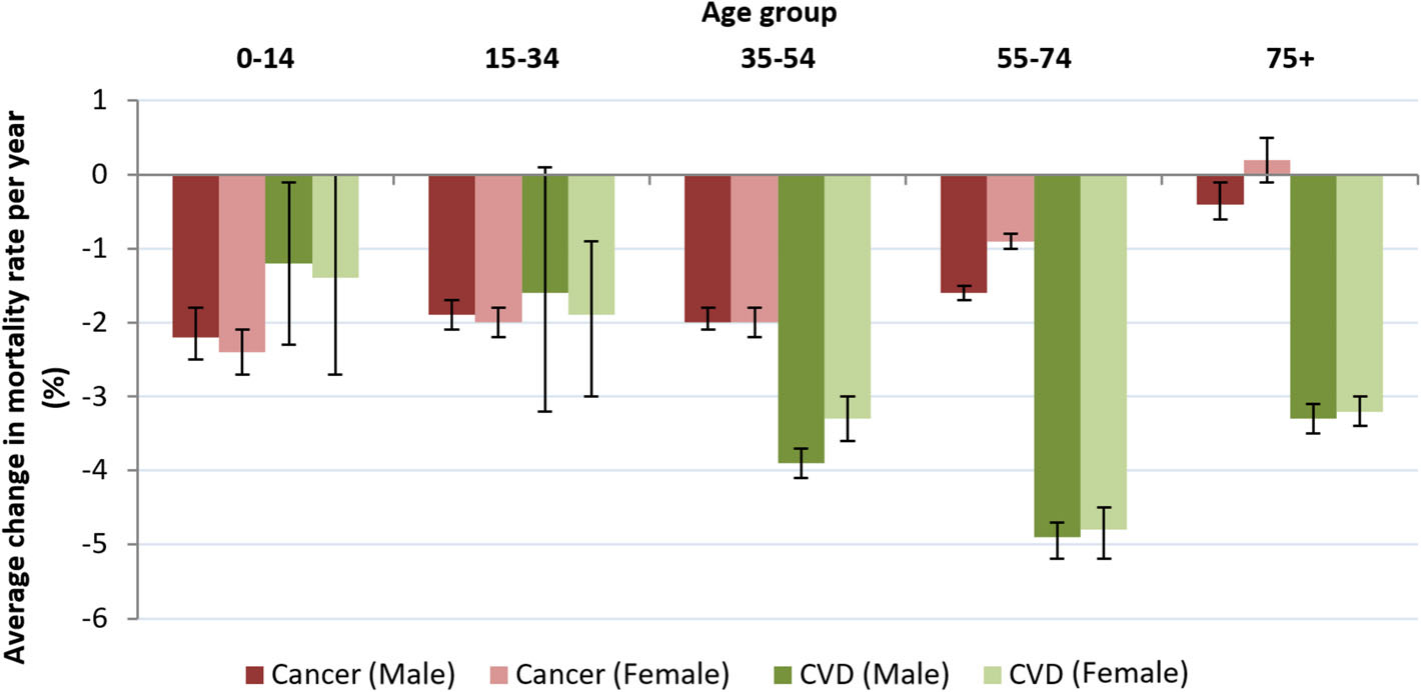
Average change in age-standardized mortality rate per year as a percentage. Legend: Years 1983 to 2013. Error bars as 95% confidence intervals

In 2013, the absolute number of deaths from CVD and cancer increased with each age group for both sexes. For the study period, most age groups showed a significant decrease in age-standardized cancer and CVD mortality rates, except cancer in women aged 75+, and CVD in men aged 15–34 years (Fig. 2). The widest sex difference in CVD mortality rate was in those aged 35–54 years. After this age group, sex differences in rate narrowed with increasing age. This pattern was not apparent for cancer, with men showing higher cancer rates than women for all age groups except those aged 35–54 years (Fig. 2).

**Fig. 3:**
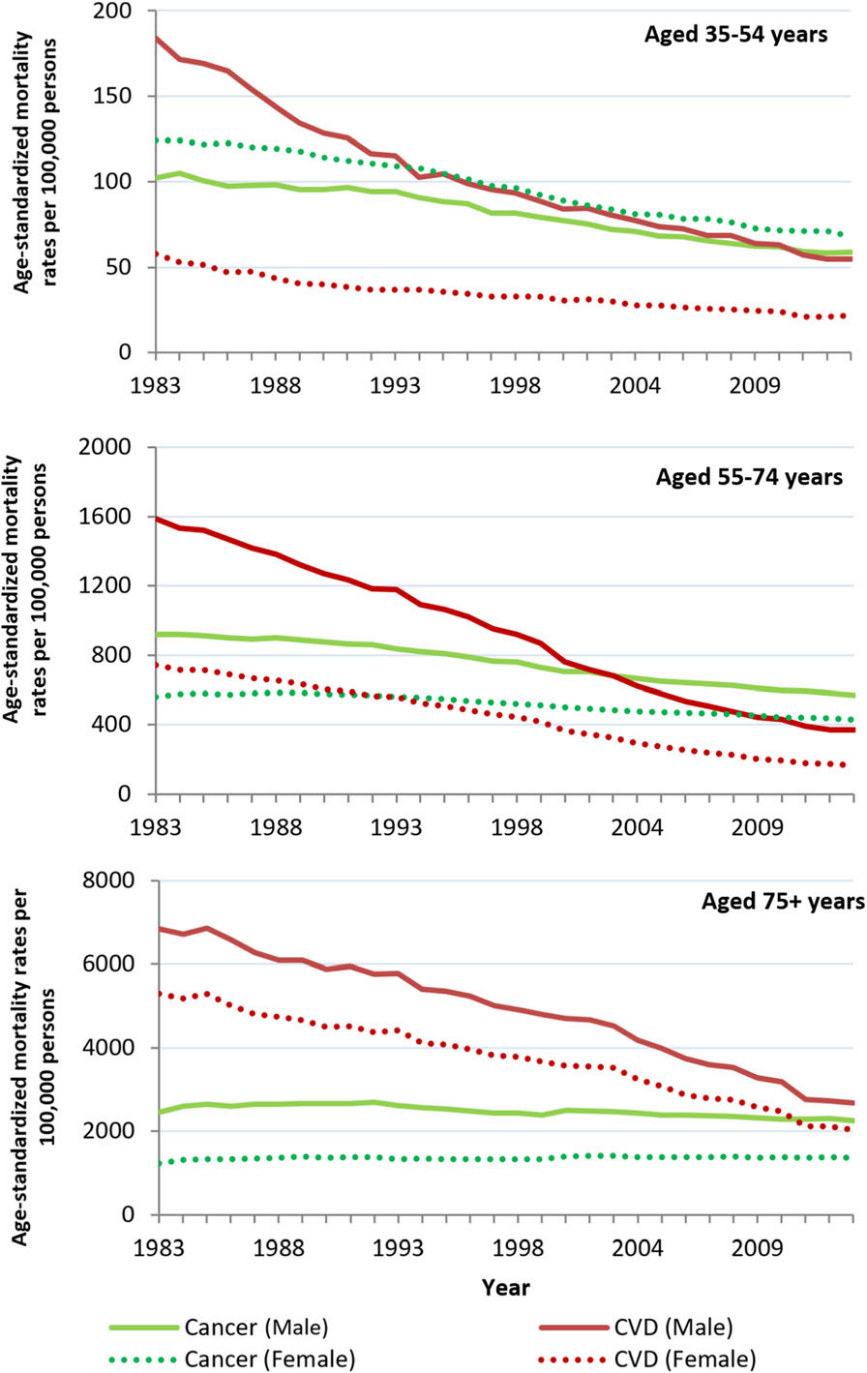
Age-standardized mortality trends for cancer and CVD by age group and sex. Legend: Years 1983 to 2013. Note different scale axis

Age-specific mortality rates showed cancer remained the most common cause of death among women less than 55 years of age and men less than 35 years. CVD remained the most common cause of death for men and women 75 years and older for the study period. Therefore the change from CVD to cancer as the primary cause of age-standardized mortality occurred in three groups: men aged 35–54 years in 2011, men aged 55–74 years in 2003, and women aged 55–74 years in 1992 (Fig. 3).

Joinpoint regression analysis showed the steepest decreases in age-specific cancer mortality rates for the study period were for men and women under 55 years (Table 3). No joinpoints were identified for cancer in men and women under 35 years. Cancer trends in age groups over 35 years were variable, with most showing a slowing in rate decrease in the most recent period.

**Table 3:** Joinpoint analysis of age-standardised^b^ mortality persons from cancer and cardiovascular diseases by age group

Age Group (years)	Total study period^c^		Period 1		Period 2		Period 3		Period 4		Period 5	
	Average APC (%)	95%CI	Years	APC (%)	Years	APC (%)	Years	APC (%)	Years	APC (%)	Years	APC (%)
Cancer												
Male												
0–14	−2.2^a^	−2.5, −1.8	−									
15–34	−1.9^a^	−2.1, −1.7	−									
35–54	−2.0^a^	−2.1, −1.8	1983–1993	−1.0^a^	1993–2013	−2.4^a^						
55–74	−1.6^a^	−1.7, −1.5	1983–1991	−0.7^a^	1991–2005	−2.1^a^	2005–2013	−1.7^a^				
75+	−0.4^a^	−0.6, −0.1	1983–1985	3.1^a^	1985–1992	0.2	1992–1998	−1.7^a^	1998–2002	0.6	2002–2013	−0.9^a^
Female												
0–14	−2.4^a^	−2.7, −2.1	−									
15–34	−2.0^a^	−2.2, −1.8	−									
35–54	−2.0^a^	−2.2, −1.8	1983–1988	−0.9^a^	1988–1994	−1.8^a^	1994–2003	−2.7^a^	2003–2013	−2.0^a^		
55–74	−0.9^a^	−1.0, −0.8	1983–1985	1.7^a^	1985–1991	−0.1	1991–2003	−1.5^a^	2003–2013	−1.2^a^		
75+	0.2	−0.1, 0.5	1983–1989	1.5^a^	1989–1998	−0.5^a^	1998–2002	1.5	2002–2013	−0.3^a^		
Cardiovascular Disease												
Male												
0–14	−1.2^a^	−2.3, −0.1	1983–1995	2.8^a^	1995–2013	−3.7^a^						
15–34	−1.6	−3.2, 0.1	1983–1991	−3.0^a^	1991–1995	2.3	1995–1999	−4.7^a^	1999–2003	3.9	2003–2013	−2.8^a^
35–54	−3.9^a^	−4.1, −3.7	1983–1994	−4.9^a^	1994–2013	−3.4^a^						
55–74	−4.9^a^	−5.2, −4.4	1983–1993	−3.2^a^	1993–1998	−4.6^a^	1998–2013	−6.2^a^				
75+	−3.3^a^	−3.5, −3.1	1983–2003	−2.2^a^	2003–2013	−5.2^a^						
Female												
0–14	−1.4^a^	−2.7, −0.0	1983–1995	1.5	1995–2013	−3.2^a^						
15–34	−1.9^a^	−3.0, −0.9	1983–1989	−4.2^a^	1989–1995	2.5	1995–2013	−2.6^a^				
35–54	−3.3^a^	−3.6, −3.0	19,831,990	−5.2^a^	1990–2002	−2.1^a^	2002–2013	−3.4^a^				
55–74	−4.8^a^	−5.2, −4.5	1983–1989	−2.6^a^	1989–1998	−3.8^a^	1998–2011	−6.8^a^	2011–2013	−3.4		
75+	−3.2^a^	−3.4, −3.0	1983–2003	−2.2^a^	2003–2013	−5.2^a^						

^a^ Significantly different from zero (*p* < 0.05)

^b^ Standardised to the 2013 European standard population by 5 year age groups

^c^ Years 1983 to 2013

- No joinpoints identified

*APC*, annual percent change

The steepest decreases in CVD mortality rate for the study period were for men and women aged 55–74 years, followed by those aged 35–54 years (Table 3). In men over 54 years, the decrease in age-standardized CVD mortality increased with each period identified. This was the same for women except for no significant change in the most recent period for women aged 55–74 years (years 2011–2013). In men and women aged 15–54 years, the steepest decreases were observed in the first period followed by non-significant periods or a slowing in rate. While in women of this age, the CVD mortality rate improved in the latest period, this has yet to occur for men of this age group.

### Disease type mortality trends

**Fig. 4:**
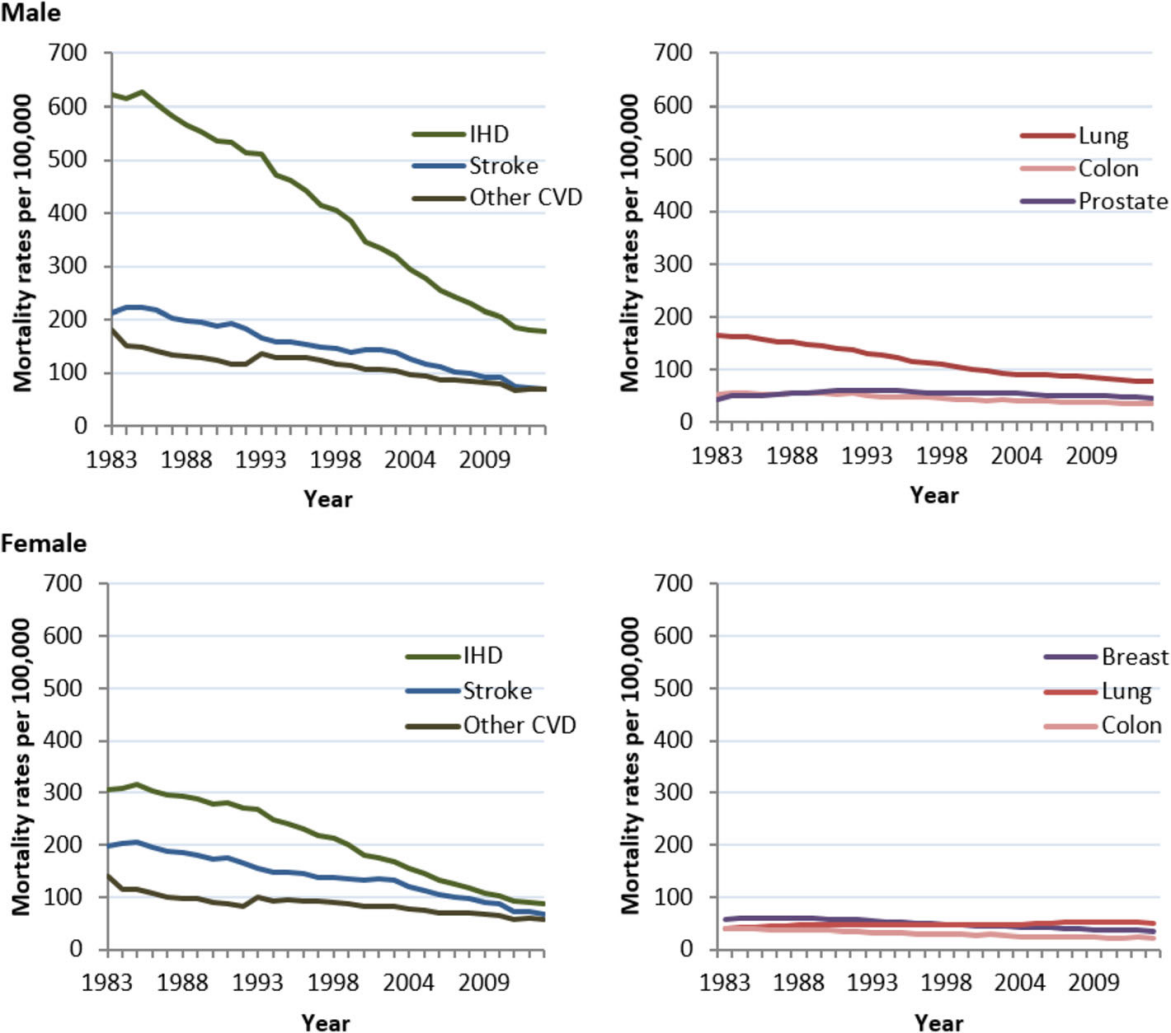
Age-standardized mortality trends for IHD, stroke, other CVD, and lung, colon, breast, and prostate cancer. Legend: Years 1983 to 2013. IHD: ischemic heart disease

The absolute number of deaths over the study period increased for prostate cancer, female lung cancer, and slightly for male colorectal cancer, while absolute deaths of all CVD types decreased. Of the types considered, IHD had the highest age-standardized mortality rate for CVDs (and overall mortality) for both sexes for the study period. Men and women had similar rates of stroke and other CVD but men had much higher rates of IHD. For cancer, the highest age-standardized mortality rate for both sexes was for lung cancer, although this trend occurred starting in 1998 for women (Fig. 4). Most disease types had significant decreases in age-standardized mortality from 1983 to 2013. The exceptions were female lung cancer, with a small significant increase, and prostate cancer, which had no significant change in overall mortality rate.

Joinpoint regression analysis showed that the steepest decreases in CVD type for the period were for IHD at a decrease of 4.1% per year for both sexes (Table 4). The decrease in age-standardized rate of IHD and stroke increased with each period identified for both sexes, except for a period with no significant change in IHD from 2011 to 2013. Other CVD saw its steepest decreases in the first period identified, followed by no significant change, and a slowing in rate in the most recent period.

**Table 4:** Joinpoint analysis of age-standardised mortality^b^ rates by leading types of cancer and cardiovascular diseases

	Mortality per 100,000		Total study period^c^		Period 1		Period 2		Period 3		Period 4		Period 5	
	1983	2013	Average APC (%)	95%CI	Years	APC (%)	Years	APC (%)	Years	APC (%)	Years	APC (%)	Years	APC (%)
Cancer														
Lung														
Male	165.7	77.2	−2.6^a^	−2.8, −2.4	1983–1991	−2.0^a^	1991–1997	−3.8^a^	1997–2004	−2.9^a^	2004–2008	−1.1^a^	2008–2013	−2.5^a^
Female	39.9	50.7	0.7^a^	0.5, 1.0	1983–1989	2.4^a^	1989–2004	0.1	2004–2008	2.1^a^	2008–2013	−0.5		
Colorectal														
Male	54.0	35.3	−1.5^a^	−1.9, −1.0	1983–1992	−0.3	1992–1995	−3.7	1995–2013	−1.6^a^				
Female	38.9	22.8	−2.1^a^	−2.2, −1.9	1983–2013	−2.1^a^								
Prostate														
Male	43.7	46.8	0.1	−0.1, 0.3	1983–1992	3.0^a^	1992–2013	−1.2^a^						
Breast														
Female	56.2	35.5	−1.5^a^	−1.8, −1.2	1983–1985	2.6	1985–1991	−0.4	1991–1998	−2.9^a^	1998–2002	−1.3	2002–2013	−2.1^a^
Cardiovascular Diseases														
Ischaemic Heart Disease														
Male	622.1	177.2	−4.1^a^	−4.5, −3.7	1983–1985	−0.2	1985–1993	−2.5^a^	1993–2002	−4.4^a^	2002–2011	−6.2^a^	2011–2013	−3.3
Female	306.2	86.5	−4.1^a^	−4.5, −3.8	1983–1985	1.0	1985–1993	−2.0^a^	1993–2003	−4.5^a^	2003–2011	−7.0^a^	2011–2013	−3.8
Stroke														
Male	213.4	70.0	−3.7^a^	−4.5, −2.9	1983–1985	2.0	1985–1998	−3.4^a^	1998–2002	0.3	2002–2013	−6.5^a^		
Female	199.1	67.9	−3.5^a^	−4.1, −3.0	1983–1985	1.1	1985–1997	−3.2^a^	1997–2003	−1.0	2003–2013	−6.3^a^		
Other CVD														
Male	180.2	69.0	−2.9^a^	−3.7, −2.1	1983–1991	−4.4^a^	1991–1995	3.6	1995–2013	−3.6^a^				
Female	140.0	58.1	−2.6^a^	−3.5, −1.7	1983–1991	−5.2^a^	1991–1995	3.8	1995–2013	−2.8^a^				

^a^Significantly different from zero (*p* < 0.05)

^b^ Standardised to the 2013 European standard population by 5-year age groups

^c^ Years 1983 to 2013

*APC*, annual percent change

Compared to CVD, trends in cancer mortality rates had fewer similarities between types, even between sexes, with a tendency toward fluctuating rates of mortality decrease and periods of non-significant change in rate. The largest overall decreases in cancer were for lung cancer in men and colorectal cancer in women. While male lung cancer was characterized by five periods of decreasing mortality at variable rates, female lung cancer showed no periods of decrease.

## Discussion

The results of this study support the evidence for decreasing mortality rates of CVD [2, 12] and cancer [13] in the UK, expanding the analysis over 30 years with a comparison of the two diseases. Mortality rates were higher for CVD than cancer for most of the study period, with a change to cancer having the higher age-standardized mortality rate for both sexes in 2011. There was a narrowing of the difference in mortality between CVD and cancer by most sex, age, and disease-type groups. This appears to be because of a steeper decrease in mortality from CVD and a slowing in the decrease of cancer since 1997.

The decrease in CVD and cancer mortality observed in the UK has been seen in most other countries of Western Europe. This includes a small decrease of the most common cancers [4, 14], increasing female lung cancer [4], and a larger decrease in CVD mortality [3, 15]. The change from CVD to cancer as the more common cause of age-standardized mortality in both sexes has occurred in Denmark, France, Israel, and the Netherlands, and in Belgium, Portugal, and Spain for males only [5]. Further analysis is required to determine if the underlying trends as observed in this study are occurring in other European countries.

Mortality rates have decreased for CVD due to improvements in risk factors and progress in diagnosis and medical treatment, including acute hospital cases [16–19]. For example, uptake of treatments, such as statins and hypertensive medicines, was attributed to half of the decrease in IHD mortality between 2000 to 2007 in England [16, 20]. Modest decreases in colon, breast, and prostate cancer were attributed to advances in treatment and earlier diagnosis [13, 21]. The effectiveness of screening is debated due to lag times [22]. For example, the outcomes of introducing bowel cancer screening in the UK in 2006 are unclear [23]. In the UK, the decrease in smoking attribution to cancer in men is far larger than in women, likely due to the delayed habit uptake from women in the 1940s [24].

The Global Burden of Disease 2015 suggests only 47% of cancer mortality rates were related to modifiable risk factors compared to 80% of CVD mortality rates in the UK [25]. Although cancer and CVD share many risk factors, a change in risk prevalence does not contribute to an equal change in cancer and CVD mortality over time. For example, the overall prevalence of smoking has decreased over 25 years, but the improvement in smoking-attributed mortality rate has been greater for CVD than cancer. This is likely due to the shorter lag effect from smoking exposure to disease for CVD. To assess the etiology of the trends determined in this paper, further research will need to consider the differing effects of risk factors on cancer and CVD over time.

Assuming current trends in risk reduction, modeling studies predict IHD death rates to continue declining in the UK [26]. Yet high body mass index (BMI) is a risk factor for CVD [27] and some cancers [28]. In England, the prevalence of obesity and associated diabetes is increasing, particularly in young women and those already with a high BMI [29, 30]. A study of English diabetics suggested there had been little decrease in IHD mortality in this group between 1995 and 2010 [31]. Previous reports from England and the US suggest a slowing in the decrease of CVD mortality in those aged 35–54 years during the 1990s with links to rising obesity [6, 32]. Our study suggested decreases in CVD mortality have steepened in women of this age in the last decade, a trend also observed in the Netherlands [33], but so far not in men. Continuing to monitor trends is essential to understand the effect of these interactions on public health.

### Limitations

The UK mortality data derived from the WHO database are considered to be of high quality due to high death registration coverage of its constituent countries and low levels of ICD miscoding [34]. Coding rules are in place to ensure that the correct underlying cause of death is selected with the majority of deaths coded automatically with software. However, mortality from CVD can be difficult to assess by death certificates [35]. For those over the age of 75 years, there is a higher chance of coexisting conditions with CVD, particularly in cancer survivors [36], increasing the difficulty of determining a single cause of death. One study in England found that considering only the underlying cause of death resulted in missing around a quarter of all stroke deaths between 1979 and 2004 [37]. Coding revision from ICD-9 to ICD-10 was known to affect records of stroke and other CVD in the 1980s to 1990s by a temporary use of only the underlying cause of death on certificates [38]. In addition, ICD coding reassignment caused some disease categories to increase in number of deaths, including a 2.3% increase in numbers of malignant neoplasms [39] and a 3%–4% increase in circulatory diseases [38]. Therefore, the results from the initial period of our study period may show unintended variation. The low number of cases at younger ages can create a less reliable age-standardized rate.

## Conclusions

The UK saw a narrowing in age-standardized mortality rates from CVD and cancer for both sexes between 1983 and 2013. Both diseases significantly decreased overall except for female lung cancer, which significantly increased during this period. Rates of decrease have slowed for cancer in both sexes.

## Abbreviations

AAPC: Average annual percentage change
APC: Annual percentage change
ASMR: Age-standardised mortality rate
BMI: Body mass index
CVD: Cardiovascular disease
ESP: European standard population
ICD: International classification of diseases
IHD: Ischemic heart disease
